# Profiling of the fecal microbiota and circulating microRNA-16 in IBS subjects with *Blastocystis *infection : a case–control study

**DOI:** 10.1186/s40001-023-01441-8

**Published:** 2023-11-06

**Authors:** Alireza Olyaiee, Abbas Yadegar, Elnaz Sadat Mirsamadi, Amir Sadeghi, Hamed Mirjalali

**Affiliations:** 1 Department of Biology, Science and Research Branch, Islamic Azad University, Tehran, Iran; 2 Foodborne and Waterborne Diseases Research Center, Research Institute for Gastroenterology and Liver Diseases, Shahid Beheshti University of Medical Sciences, Tehran, Iran; 3 Department of Microbiology, Faculty of Medicine, Tehran Medical Sciences, Islamic Azad University, Tehran, Iran; 4 Gastroenterology and Liver Diseases Research Center, Research Institute for Gastroenterology and Liver Diseases, Shahid Beheshti University of Medical Sciences, Tehran, Iran

**Keywords:** IBS, *Blastocystis*, Fecal microbiota, MicroRNA, mir-16

## Abstract

Irritable bowel syndrome (IBS) is a prevalent gastrointestinal (GI) tract disorder. Although the main reason for IBS is not clear, the interaction between intestinal microorganisms and the gut barrier seems to play an important role in pathogenesis of IBS. The current study aimed to investigate the effect of *Blastocystis* on the gut microbiota profile and the circulation levels of microRNA (mir)-16 of IBS patients compared to healthy subjects. Stool and blood samples were collected from 80 participants including 40 samples from each IBS and healthy group. Upon DNA extraction from stool samples, barcoding region and quantitative real-time PCR were analyzed to investigate *Blastocystis* and the microbiota profile, respectively. RNA was extracted from serum samples of included subjects and the expression of mir-16 was evaluated using stem-loop protocol and qreal-time PCR. Significant changes between IBS patients and healthy controls was observed in Firmicutes*,* Actinobacteria*, Faecalibacterium,* and *Alistipes*. In IBS patients, the relative abundance of *Bifidobacteria* was directly correlated with the presence of *Blastocystis*, while *Alistipes* was decreased with *Blastocystis*. *Lactobacillus* was significantly increased in *Blastocystis* carriers. In healthy subjects, the relative abundance of *Bifidobacteria* was decreased, but *Alistipes* was increased in *Blastocystis* carriers. The changes in the Firmicutes/Bacteroidetes ratio was not significant in different groups. The relative expression of mir-16 in *Blastocystis*-negative IBS patients and healthy carriers was significantly overexpressed compared to control group. The presence of *Blastocystis*, decreased the relative expression of mir-16 in IBS patients compared to *Blastocystis*-negative IBS patients. The present study revealed that *Blastocystis* has the ability to change the abundance of some phyla/genera of bacteria in IBS and healthy subjects. Moreover, *Blastocystis *seems to  modulate the relative expression of microRNAs  to control the gut atmosphere, apply its pathogenicity, and provide a favor niche for its colonization.

## Introduction

Irritable bowel syndrome (IBS) is one of the most common functional disorders of the digestive system [1, 2]. The pathophysiological mechanisms behind the IBS have not been cleared yet; however, the main symptoms are abdominal bloating, visceral pain, and changes in the stool pattern [3]. IBS is a heterogeneous disorder with four main types: IBS-C with constipation stool type, IBS-D with diarrhea stool type, IBS-M with intermittent bowel pattern, and IBS-U with not-classified pattern of stool [4]. This disease affects about 10–20% of the world's population [5]. It was estimated that about 20% of the population and more than half of the visits to gastroenterology clinics in the UK complain from IBS symptoms, with a higher prevalence among women [6]. It is believed that the genetic, psychological and social factors, motility changes in the digestive system, increased visceral sensitivity, gut–brain axis disorders, activation of mast cells in the intestinal mucosa, and changes in serotonin metabolism play major role in the development and pathogenesis of IBS [7]. Furthermore, the interaction between microorganisms and the digestive system is thought to be involved in the progress of this disease [1]. There is evidence indicating significant role of gut microbiota disturbance in IBS patients [6]. Variation in the microbiota composition and metabolites compared to the healthy subjects highlights the role of gut microbiota in IBS [8, 9]. Therefore, the gut microbiota has emerged as a key issue in the investigation of gastrointestinal diseases such as IBS [4, 10].

The microbial community that lives in the human body (in the gut) is called the microbiota, which consists of a wide range of microorganisms including bacteria, viruses, fungi, and other eukaryotes [10]. Bacteria cover a large and eukaryotes comprise very small portion of the gut microbiota; nevertheless, protists such as *Blastocystis* may play important role in gut microbiota composition and richness [1, 11].

*Blastocystis* is a cosmopolitan protozoan in humans and animals [12], which is commonly reported from patients who suffer from IBS [13, 14]. *Blastocystis* is the most common parasite living in the human body, which affects about 5–30% of people in advanced and industrial societies and about 30–50% of people in non-industrial societies [15]. Although pathogenicity of *Blastocystis* is controversial, it was suggested that the protozoan can affect intestinal permeability [16, 17], and probably induces apoptosis throughout the gut [18].

There is a little data about the correlation of *Blastocystis* and the gut barrier functions. Recently, the role of *Blastocystis* on the regulation of micro RNAs (miRNAs/mir) and tight junction (TJ) proteins of the gut has been suggested [19]. It was shown that total antigen derived from *Blastocystis* subtype 3 induces the expression of mir-223 and mir-874, which both are associated with gut barrier dysfunctions [19]. MiRNAs are short non-coding, single-stranded RNAs that found in all eukaryotic and human cells, with an average length of 19–25 nucleotides, which control protein-coding gene expression at the post-transcriptional stage [20]. MiRNAs, for example mir-16, are considered as regulators in controlling of the expression of intestinal TJ proteins and the intestinal epithelial barrier [21]. A negative correlation was suggested between the expression levels of mir-16 and TJ proteins (cingulin and claudin-2) in IBS-D patients [21].

In the current study, we investigated the pattern of selected gut microbiota composition and mir-16 expression in IBS patients and healthy controls regarding the presence of *Blastocystis.*

## Materials and methods

### Ethics approval and consent to participate

This study received ethical approval from the Ethics Committee of the Islamic Azad University, Science and Research Branch, Tehran, Iran (IR.IAU.SRB.REC.1400.241).

Informed consent was verbally obtained from all participates and/or their legal guardian(s). For those patients with age ≤ 16, informed consent was obtained from their respective parent(s)/guardian(s) as well.

### Sample collection and DNA extraction

**Fig. 1:**
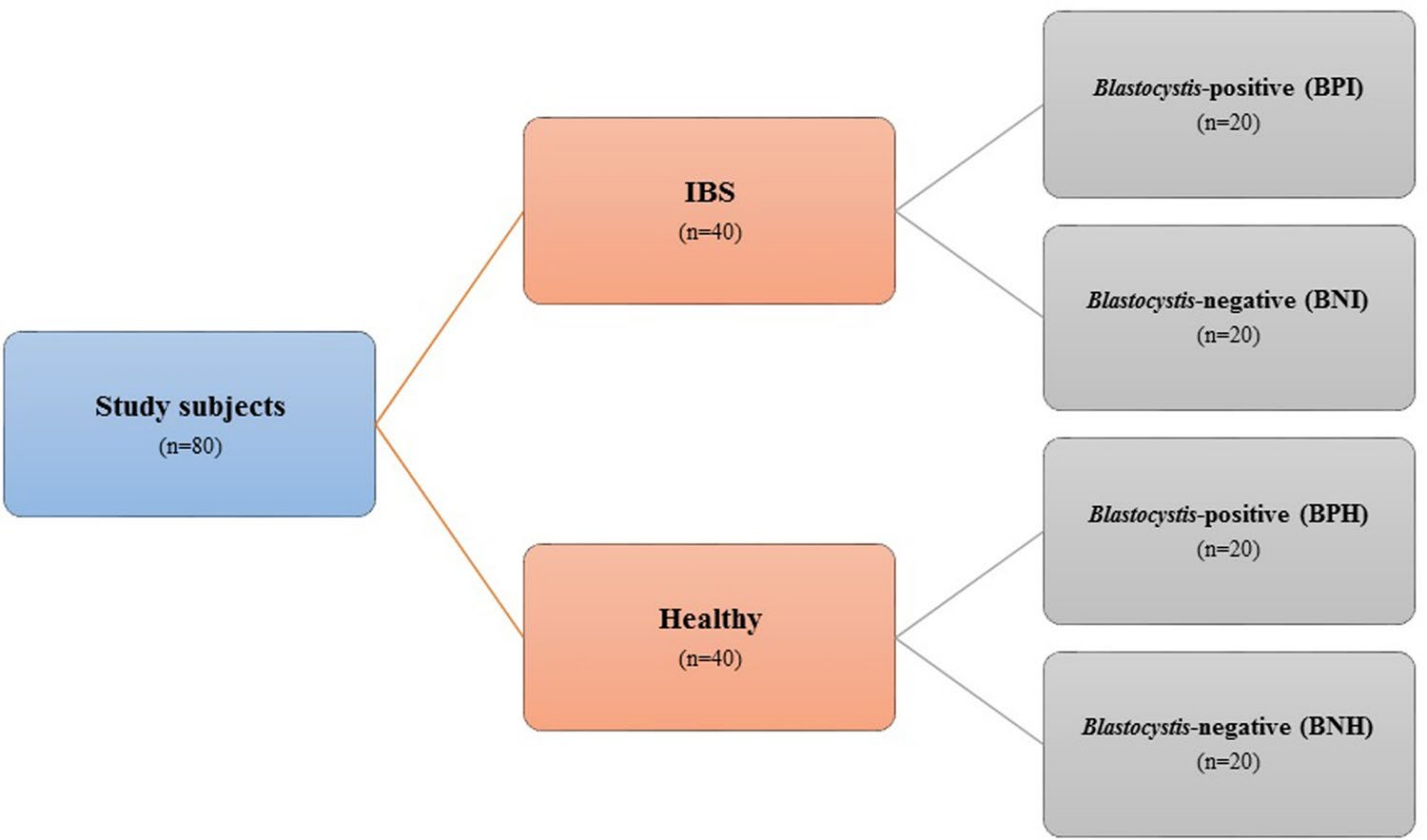
The flowchart describing allocation of samples in studied groups

Stool samples were collected from 80 participants including 40 samples from each IBS and healthy group. To analyze the effects of *Blastocystis* on the gut microbiota and mir-16 expression levels, four groups were considered for the study including 20 samples for each *Blastocystis-*positive IBS patients (BPI), *Blastocystis-*negative IBS patients (BNI), *Blastocystis-*positive healthy subjects (BPH), and *Blastocystis-*negative healthy subjects (BNH) (Fig. 1). All stool samples were examined for the presence of intestinal parasites using direct microscopy. The presence of *Blastocystis* in stool samples was confirmed using amplification of the “barcoding region” through the small subunit ribosomal RNA (*SSU rRNA*) gene [22]. The presence of any other cysts/oocysts/eggs in stool samples was considered as exclusion criteria. Healthy controls were collected from those subjects who either intend to participate in our study or referred to the laboratory for periodical checkup. Healthy controls did not complain any gastrointestinal disorders and those who suffer from any gastrointestinal problems were excluded from the study.

Demographic data, drug consumption, stool appearance at the time of sampling, and symptoms during sampling were recorded. The presence of IBS was confirmed by gastroenterologists, and consumption of any antibiotics, immunosuppressant drugs, alcohol, following a specific diet, and the presence of any immunodeficiency were considered as exclusion criteria. In addition, in healthy controls, the presence of any gastrointestinal symptoms was considered as exclusion criterion.

To analyze the expression of mir-16, peripheral blood samples were collected from all participants, as well. To analyze the expression of circulating mir-16, serum was isolated from blood samples. All samples were transferred to the Parasitology lab in the Research Institute for Gastroenterology and Liver Diseases for further analyses. Stool and serum samples were kept out at −20 and −70 ˚C, respectively, until experiments.

To characterize the presence of *Blastocystis* and selected microbiota, total DNA was extracted from stool samples using stool DNA extraction kit (Yekta Tajhiz, Tehran, Iran). For this purpose, 200 mg of stool samples was washed three times with sterile phosphate buffer saline (PBS; pH = 7.5) and after discarding supernatant, DNA was extracted from remained pellet. Isolated DNA was stored at −20 °C until use.

### Microbiota profiling

Quantitative real-time PCR was employed to investigate the microbiota profiling of a selection of phyla/classes/genera according to the literature review, including phyla Firmicutes, Bacteroidetes, Actinobacteria, and Fusobacteria, and genera *Lactobacillus*, *Bifidobacteria*, *Streptococcus*, *Faecalibacterium*, *Blautia*, *Ruminococcus*, *Roseburia*, *Alistipes*, *Prevotella*, and *Methanobacteria.* Amplification was performed using phyla- and genus-specific, and universal primers, which were mentioned (Table 1).

**Table 1:** Targeted microorganism and their primers

Microorganisms	Oligonucleotide sequence (5`–3`)	Product size (bp)	Refs
Eubacteria	ACTCCTACGGGAGGCAGCAGT	200	[25]
	ATTACCGCGGCTGCTGGC		
Firmicutes	GGAG**Y**ATGTGGTTTAATTCGAAGCA	129	[26]
	AGCTGACGACAACCATGCAC		
Bacteroidetes	GTTTAATTCGATGATACGCG	137	[26]
	TTAAGCCGACACCTCACG		
Actinobacteria	GCGACCTATCAGCTTGTT	345	[27]
	CCGCCTACGAGCTCTTTACGC		
*Ruminococcus* spp.	GGCGGC**Y**TRCTGGGCTTT	302	[28]
	CCAGGTGGAT**W**ACTTATTGTGTTAA		
*Prevotella* spp.	CACCAAGGCGACGATCA	507	[29]
	GGATAACGCCTGGACCT		
*Methanobacteria* spp.	CGATGCGGACTTGGTGTTG	184	[29]
	TGTCGCCTCTGGTGAGATGTC		
*Faecalibacterium* spp.	GATGGCCTCGCGTCCGATTAG	198	[29]
	CCGAAGACCTTCTTCCTCC		
*Bifidobacteria* spp.	GGGATGCTGGTGTGGAAGAG	200	[30]
	TGCTCGCGTCCACTATCCAG		
*Lactobacillus* spp.	TGGATGCCTTGGCACTAG	89	[31]
	AAATCTCCGGATCAAAGCTTAC		
*Roseburia* spp.	GCGGTGCGGCAAGTCTGA	81	[This study]
	CCTCCGACACTCTAGTACGAC		
*Blautia* spp.	GCAAGTCTGATGTGAAAGGCTG	251	[This study]
	TTGCCACCCGACACCTAGTA		
*Alistipes* spp.	TTAGAGATGGGCATGCGTTGT	320	[32]
	TGAATCCTCCGTATT		
Fusobacteria	GATCCAGCAATTCTGTGTG	290	[29]
	CGAATTTCACCTCTACACTTG		
*Streptococcus* spp.	GTACAGTTGCTTCAGGACGT	195	[27]

Quantitative real-time PCR was performed in total volume 15 μL using a Rotor-Gene Q (QIAGEN, Germany) real-time instrument based on the following conditions: 7.5 μL of 2X real-time PCR Master Mix (BioFACT™, Korea), 5 ρM of each primer, 3.5 μL of distilled water, and 3 μL of template DNA. The cycling profile was 95 °C for 10 min followed by 40 cycles: 95 °C for 25 s, 56 °C for 30 s and 72 °C for 20 s and ramping from 70 °C to 95 °C at 1 °Cs − 1. Positive control and sterile distillated water were run together with each sample set, as positive and negative controls, respectively. To exclude the probability of false amplification and primer-dimers, melting curve analysis was performed. In the cases of cycle of threshold (*C*_*t*_) value more than 35, amplification plot and melting profile were considered to exclude negative samples. The relative abundance of bacterial taxonomic group was calculated according to the method described elsewhere [23, 24] based on the following formula:

$$X \, = \, \left( {{\text{Eff}}.{\text{Univ}}} \right)^{C_t {\text{ univ}}} / \, \left( {{\text{Eff}}.{\text{Spec}}} \right)^{C_t {\text{ spec}}} \times {1}00.$$

The Eff. Univ represents the calculated efficiency of the universal primers for Eubacteria (2 = 100% and 1 = 0%), the Eff. Spec indicates the efficiency of the taxon-specific primers. In addition, *C*_*t*_ univ and *C*_*t*_ spec refer to the released *C*_*t*_ by the thermocycler, and “*X*” is the percentage (%) of taxon-specific *16S rRNA* gene copy numbers in single fecal sample.

### RNA extraction, cDNA synthesis, and quantitative real-time PCR

The expression level of mir-16 was evaluated using stem-loop reverse transcriptase and real-time PCR using primers and protocols, which were previously explained [19]. For this purpose, RNA was extracted using Trizol extraction protocol (BioMix) from serum samples, which were collected from enrolled participants. Briefly, 750 µL of Trizol reagent was added to 250 µL of serum. After agitating for 10 s and cooling at − 20 ˚C for 10 min, 250 µL of chloroform was added and cooled again at – 20 ˚C for 7 min. Samples were vortexed at 4 ˚C for 20 min, centrifuged at 12000 × *g*, and supernatant was collected and mixed with isopropanol alcohol. After centrifuging at 12000 × *g* in 4 ˚C for 20 min, RNA was isolated from remained pellet. The concentration of extracted RNAs was determined by NanoDrop (NanoDrop Technologies, USA) apparatus, and RNA adjustment was performed before complementary DNA (cDNA) synthesis. cDNA was constructed for mir-16 and U6, as housekeeping gene, using cDNA synthesis kit (Pars Tous, Mashhad, Iran), as explained previously [19].

Relative expression of the mir-16 in serum samples of participants were evaluated by qreal-time PCR using Rotor-Gene Q (Qiagen, Germany) in a 20 µL reaction mixture containing 10 µL SYBR Green qPCR master mix 2X (Ampliqon, Denmark), 5 ρM of each primer, and 2 µL of constructed cDNA as template. The amplification condition for mi6-16 was adjusted with previously released protocol [19]. As mentioned above, to avoid from non-specific amplification, melting curves were analyzed for each run. The relative expression of mir-16 was adjusted to housekeeping gene (U6 snRNA) and calculated comparing to *Blastocystis*-negative healthy controls [19].

### Statistical analysis

The descriptive statistics were presented as frequency and prevalence. The quantity of bacteria and levels of mir-16 were presented as mean and standard deviation (SD). The normality of distribution was tested by the Shapiro–Wilk test, and Student’s *t* test (independent and paired sample) was employed for the parametric data analysis. Mann–Whitney and Wilcoxon tests were used for non-parametric data analysis. In addition, the principal coordinate analysis (PCoA) was calculated using the same program based on the Bray–Curtis dissimilarity method. *P* value < 0.05 was considered statistically significant. REST, SPSS v.24, GraphPad Prism version 8.3.0.538, and the open-source statistical program R version 3.6.1 (R Core Team, Vienna, Austria) software were applied for data analysis. GraphPad Prism software version 8.3.0.538 was employed for visualizing data.

## Results

### Demographic data

Regardless the presence of *Blastocystis*, the mean of age ± SD in IBS patients and healthy subjects was 41.9 ± 13.02 and 40.33 ± 13.93, respectively. The gender distribution in all studied subjects was 39 (48.75%) and 41 (51.25%) for female and male, respectively (Table 2).

**Table 2:** Demographic data of participated subjects

Demographic data	IBS		Healthy	
	*Blastocystis*-positive	*Blastocystis*-negative	*Blastocystis*-positive	*Blastocystis*-negative
Gender				
Female	11	10	9	9
Male	9	10	11	11
IBS types				
IBS-D	2	0	–	–
IBS-C	0	0		
IBS-M	0	0		
IBS-U	18	20		
Stool appearance				
Formed/soft	18	20	18	18
Loose	1	0	0	2
Diarrhea/watery	1	0	1	1
Age				
≤ 20	0	0	2	1
21–40	8	13	11	8
41–60	10	6	6	8
≥ 61	2	1	1	3

### Microbiota comparisons

Real-time PCR was performed on all included samples and results of analyses showed significant changes between IBS patients and healthy controls in four phyla and genera including, Firmicutes*,* Actinobacteria*, Faecalibacterium,* and *Alistipes*, respectively. Accordingly, the relative abundance of Firmicutes*,* Actinobacteria*,* and *Faecalibacterium,* in IBS patients was 14.52 ± 3.04, 7.73 ± 1.17, and 9.75 ± 1.41, respectively, which were higher than the relative abundance of mentioned taxa in healthy subjects. In contrast the relative abundance of *Alistipes* was increased from 2.06 ± 2.58 in IBS patients to 5.13 ± 5.79 in healthy subjects.

**Fig. 2:**
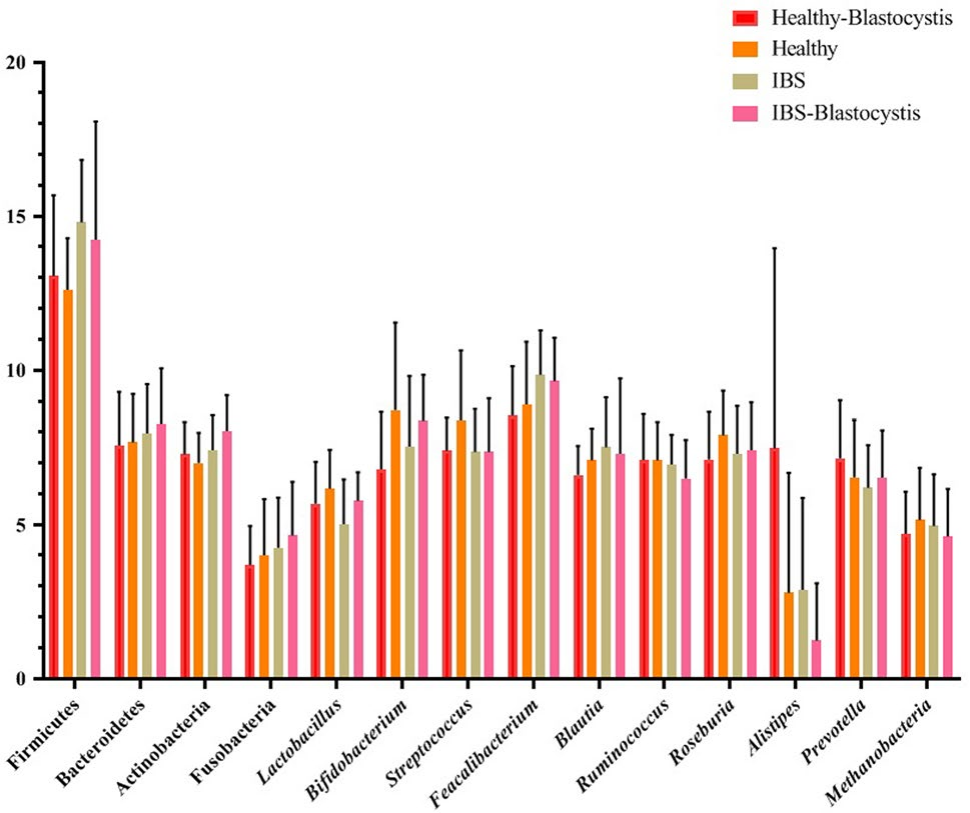
The bar-plot analysis represents the relative abundance and distribution of each targeted bacteria in studied groups as mean ± SD

Regarding the presence of *Blastocystis*, the relative abundance of *Bifidobacteria* in IBS patients was directly associated with the presence of *Blastocystis* and was increased with the protist. In contrast, the relative abundance of *Alistipes* was conversely associated with *Blastocystis*. A statistically significant change in the relative abundance of taxa was seen in *Lactobacillus*, which was increased from 5.01 ± 1.46 in *Blastocystis*-negative IBS (BNI) subjects to 5.77 ± 0.93 in BPI patients (*P *value = 0.033). In non-IBS subjects, the presence of *Blastocystis* significantly affected the relative abundance of *Bifidobacteria* and *Alistipes.* Accordingly, the relative abundance of *Bifidobacteria* was conversely related with the *Blastocystis*, and was decreased from 8.12 ± 2.61 in *Blastocystis-*negative healthy BNH to 7.58 ± 1.84 in BPH (*P *value = 0.028). In contrast, the abundance of *Alistipes* was increased from 2.84 ± 3.42 in BNH to 4.35 ± 5.67 in BPH (*P *value = 0.000) (Table 3; Fig. 2).

**Table 3:** The relative abundance of the gut microbiota in four studied groups

Taxa	IBS		Healthy		*P-*values
	*Blastocystis*-positive	*Blastocystis*-negative	*Blastocystis*-positive	*Blastocystis*-negative	
Phylum					
Firmicutes	14.238 ± 3.836	14.799 ± 2.01	13.053 ± 2.62	12.608 ± 1.68	0.01
Bacteroidetes	8.268 ± 1.79	7.958 ± 1.59	7.571 ± 1.72	7.674 ± 1.56	0.421
Actinobacteria	8.036 ± 1.16	7.423 ± 1.11	7.275 ± 1.05	6.999 ± 0.98	0.022
Fusobacteria	4.651 ± 1.73	4.253 ± 1.62	3.687 ± 1.26	4.013 ± 1.25	0.397
Genus					
*Lactobacillus*	5.772 ± 0.93	5.013 ± 1.45	5.685 ± 1.35	6.166 ± 1.25	0.029
*Bifidobacteria*	7.534 ± 2.28	8.364 ± 1.48	6.796 ± 1.84	8.702 ± 2.84	0.059
*Streptococcus*	7.364 ± 1.73	7.360 ± 1.39	7.394 ± 1.07	8.381 ± 2.26	0.472
*Feacalibacterium*	9.646 ± 1.40	9.850 ± 1.44	8.531 ± 1.60	8.893 ± 2.03	0.047
*Blautia*	7.301 ± 2.43	7.509 ± 1.61	6.612 ± 0.94	7.086 ± 1.24	0.249
*Ruminococcus*	6.500 ± 1.24	6.955 ± 0.95	7.097 ± 1.48	7.088 ± 1.24	0.324
*Roseburia*	7.423 ± 1.53	7.297 ± 1.54	7.114 ± 1.53	7.905 ± 1.43	0.233
*Alistipes*	1.238 ± 1.85	2.889 ± 2.97	7.472 ± 6.49	2.788 ± 3.89	0.000
*Prevotella*	6.527 ± 1.52	6.192 ± 1.39	7.153 ± 1.87	6.537 ± 1.85	0.322
*Methanobacteria*	4.622 ± 1.54	4.962 ± 1.67	4.704 ± 1.36	5.153 ± 1.68	0.678

### Firmicutes/Bacteroidetes ratio

**Fig. 3:**
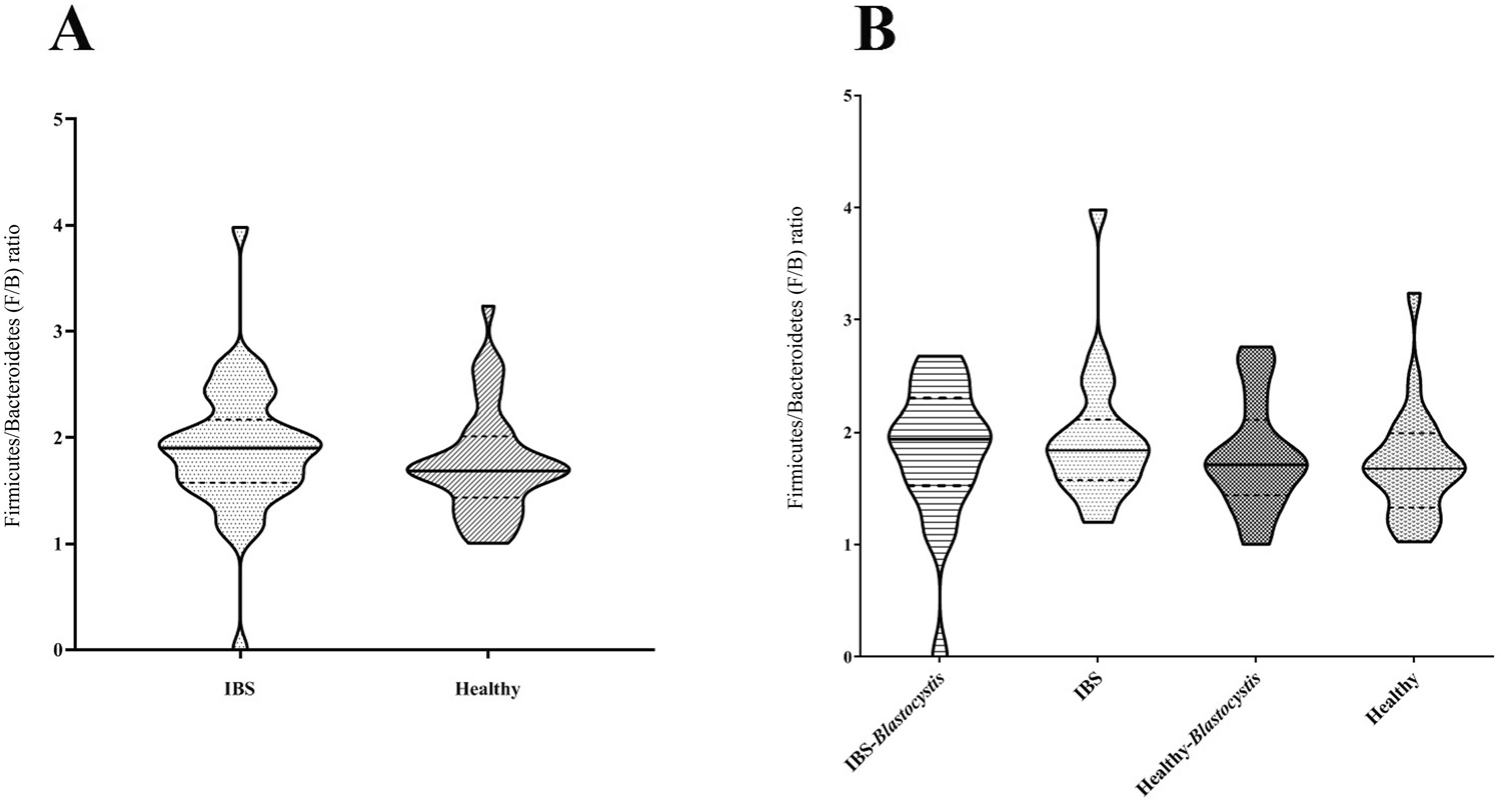
The Firmicutes to Bacteroidetes (F/B) ratio in fecal samples of **A** IBS patients and healthy controls, and **B** IBS patients and healthy controls based on the presence of *Blastocystis*. Statistical analyses based on Mann–Whitney test showed no significant association

To evaluate the hemostasis condition of the gut microbiota in studied groups, the Firmicutes/Bacteroidetes ratio was evaluated. The analysis showed a similar violin shape of the ratio in groups, while there were no significant differences in calculated ratio between IBS and healthy controls, regardless the presence of *Blastocystis*. The presence of *Blastocystis* changed the violin shape of Firmicutes/Bacteroidetes ratio in different groups, but the differences were not statistically significant (Fig. 3).

### Principal coordinate analysis

**Fig. 4:**
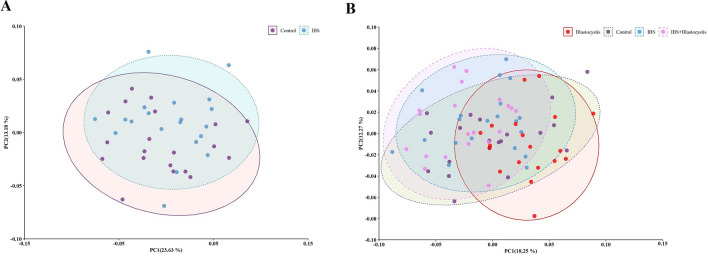
The PCoA analysis represented a comparison between the microbial communities from **A** IBS and healthy controls **A** without and **B** with considering the presence of *Blastocystis*. The comparison of groups showed a difference between healthy *Blastocystis* carriers with other three groups

Diversity of studied microbiota was evaluated by the PCoA analysis. The PCoA suggested a similar diversity in investigated taxa, regardless the presence of *Blastocystis*. The comparison of groups to each other represented a similarity through the diversity of microbiota among three groups BNH, BNI patients, and BPI patients, while they were different from healthy carriers (Fig-PCoA) (Fig. 4A, B).

### Relative expression of mir-16

**Fig. 5:**
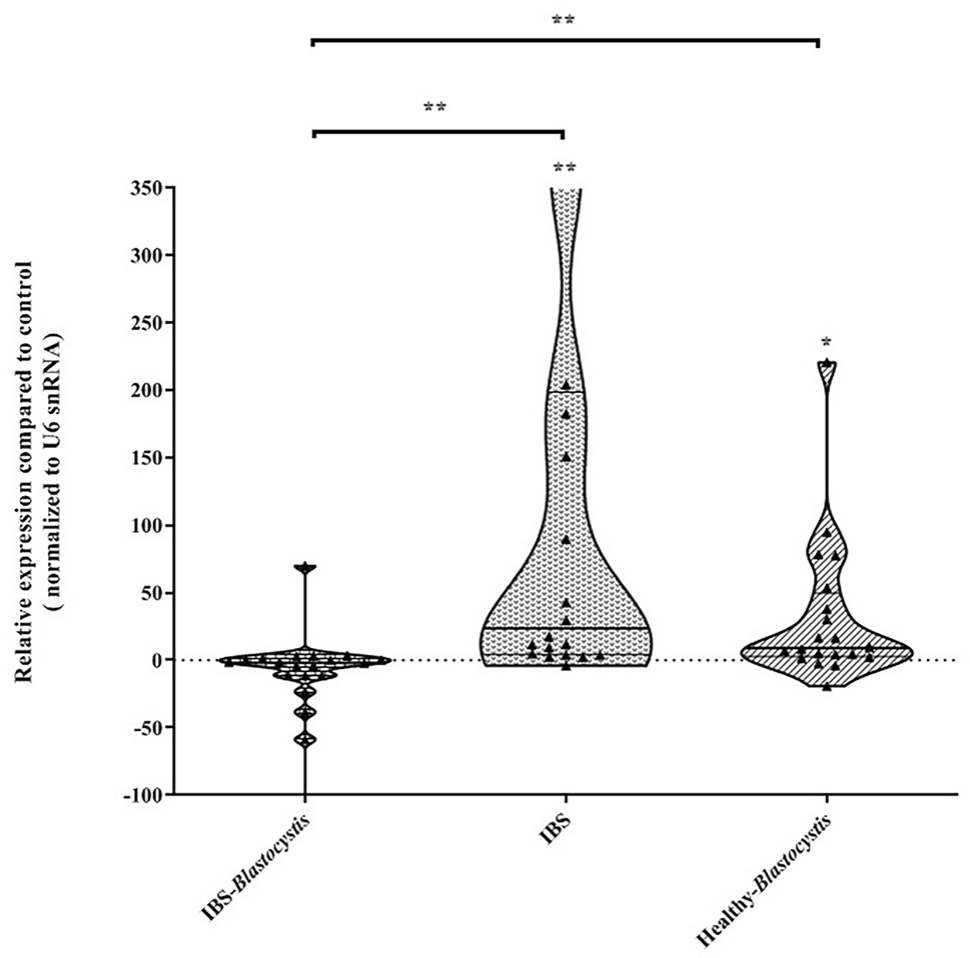
Comparison of the relative expression of circulating mir-16 in studied groups compared with *Blastocystis*-negative healthy subjects. Independent *t* test was employed to analyze statistical association. The analysis suggests a modulatory role of *Blastocystis* on the expression levels of mir-16 in IBS patients. *P value < 0.05; **P value < 0.01

The relative expression of mir-16 revealed non-significant downregulation in BPI group (16.80 ± 59.70; *P* value = 0.191) compared to BNH. The relative expression of mir-16 in BNI patients and BPH was significantly overexpressed compared to control group (120.3 ± 163; *P* value = 0.0023) and (32.17 ± 54.13; *P* value = 0.014), respectively. In IBS patients, the presence of *Blastocystis*, significantly changed the relative expression of mir-16. Accordingly, the presence of *Blastocystis*, decreased the relative expression of mir-16 from 120.3 ± 163 to 16.80 ± 59.70 (*P *value = 0.0011) (Fig. 5).

## Discussion

Alongside with increasing studies on the distribution of *Blastocystis*, many reports have indicated a high prevalence of this protist in IBS patients. However, the association between the presence of IBS and carrying *Blastocystis* is still unclear. IBS is a multifactorial disease of the GI tract, which is associated with the gut microbiota disturbance [33, 34]. Moreover, correlation between colonization of *Blastocystis* and the richness and diversity of the gut microbiota has been evaluated [35–37]. On the other hand, correlation between *Blastocystis* and the gut permeability has complicated IBS-*Blastocystis* associations [19, 38–40]. In the current study, we investigated correlation between *Blastocysts*, with a couple of bacterial taxa and the serum levels of mir-16 in IBS patients compared to healthy controls.

As a finding, regardless the presence of *Blastocystis*, the relative abundance of Firmicutes, Actinobacteria, and *Faecalibacterium* in IBS patients was significantly higher than healthy controls, which was in contrast with *Alistipes.* The richness of Firmicutes in IBS patients compared to healthy controls is controversial. Although available evidence supports our results, which indicate higher abundance of Firmicutes in IBS patients [8], many studies have demonstrated lower richness of Firmicutes in IBS patients compared to control subjects [2, 41, 42]. This contentious trend was also observed for the abundance of Actinobacteria, and *Faecalibacterium* [2, 8, 43, 44]. However, this controversial results most probably backs to the method of investigations and IBS types [1]. For example, Zhuang et al. [34], demonstrated that the abundance of Firmicutes was decreased in IBS-D patients, while Bacteroidetes was increased. Nevertheless, in the line of previous studies [8, 45], the relative abundance of *Alistipes* in IBS patients was decreased in comparison to healthy subjects.

*Alistipes* is commonly isolated from healthy gut, however, there are contradicting reports of some species such as *A. obesi* and *A. ihumii* in morbidly obese and anorexia patients, respectively [46]. It was suggested that *Alistipes* is associated with short chain fatty acids (SCFAs) and could be an acetate and propionate producer [47, 48]. Moreover, SCFAs significantly contribute in pathophysiology and severity of IBS [49]. Butyrate, acetate, and propionate levels in stool are conversely related with IBS-C, while it is positively associated with IBS-D [50, 51]. In the current study, diarrhea was not seen in most of participated IBS patients, therefore, higher and lower abundance of Firmicutes and *Alistipes* in our samples compared to healthy controls could be rational. Splitting IBS and healthy groups based on *Blastocystis,* revealed a decreased abundance of *Bifidobacterium* and *Alistipes* in BPH and BNH controls, respectively. However, *Lactobacillus* and *Bifidobacterium* were enriched in BPI patients. *Lactobacillus*, *Alistipes*, and *Bifidobacterium* are SCFAs-producing bacterial genera [47, 52]. On the other hand, enrichment of SCFAs-producing bacteria was documented to be correlated with *Blastocystis* subtypes. For example, Deng et al. [53] demonstrated a positive association between *Blastocystis* subtype 4 with SCFAs-producing bacteria, while this correlation was converse for subtype 7. Controversial correlation between *Blastocystis* subtypes and enrichment of SCFAs-producing bacteria was supported by other studies [54, 55]. In the current study, *Blastocystis* -positive subjects were probably consisted of a diverse subtype pattern. Therefore, conflicting correlation between enrichment of SCFAs-producing bacteria and the presence of *Blastocystis* could be related to not only the gut conditions, like IBS, but also different subtypes.

The intestinal barrier plays an important role in maintaining and balancing intestinal homeostasis, and prevents the transfer of contents from the lumen to the lower layer and the circulatory system [56]. Therefore, the disturbance in the functions of the intestinal barrier can cause disturbances in the immune system of the GI tract [56, 57]. TJ proteins are responsible for the integrity of the intestinal barriers, and the presence of disorder in these proteins affects the intercellular permeability [56]. In addition, it is believed that the permeability of the intestinal barrier is regulated by miRNAs [58, 59]. Martinez et al. [21] demonstrated a significant reverse association between the mir-16 and TJ proteins, caludin-2 and cingulin, in IBS-D patients. They reported that the number of mast cells was negatively correlated with has-mir-16 and has-mir-125b, while it was positively associated with caludin-2 and cingulin, highlighting the significant role of miRNAs in expression of TJ proteins and the severity of clinical symptoms in IBS patients [21]. The protecting role of mir-16 in IBS-D patients was supported by Xi et al. [60], who showed that mir-16 may inhibit TLR4/NF-κB pathway signaling, leading to prevention from apoptosis and stimulation of enterocyte viability. In a single study, evaluating the effects of *Blastocystis* on the expression of miRNAs, Mohammad Rahimi et al. [19], suggested significant effects of *Blastocystis* soluble total antigen (B3STA), extracted from subtype 3, on the expression of a couple of miRNAs (mir-223, mir-874, and mir-29a) and claudin-7, which all are contributed to the gut barrier integrity. They showed that although B3STA decreased the expression of mir-29a, it induced the expression of mir-223, mir-874, which are all involved in the dysfunction of the gut barrier. In the current study, the circulating level of mir-16 in BPI patients was significantly lower than BNI group, and was similar to BNH subjects. Our results suggested that *Blastocystis* may induce inflammation and elevate the gut permeability via dysregulating miRNAs in IBS patients, while the association between *Blastocystis* and miRNAs could be different in healthy controls. However, colonization of *Blastocystis* in the gut may either directly or indirectly induce inflammation and affects the gut integrity via changing the microbiota, in a non-healthy condition, like IBS. Although there are studies describing the effects of *Blastocystis* on the gut microbiota, this is the first study analyzing the effects of this protist on not only the gut microbiota, but also the transcriptional expression of mir-16, which is involved in the gut permeability in IBS patients. However, the most important limitation of this study is the lack of metagenomics data, which can provide much more data about the microbiome of the gut in IBS patients. In addition, the lack of a microarray analysis to highlight a table of miRNAs, which are affected by *Blastocystis*, is another limitation, that are recommended to be considered in future studies.

## Conclusion

The present study revealed that although the presence of *Blastocystis* was not significantly associated with the Firmicutes/Bacteroidetes ratio, this protozoan decreased the abundance of *Bifidobacterium* and *Alistipes* in healthy controls, while in IBS patients, *Lactobacillus* and *Bifidobacterium* were enriched in *Blastocystis* carriers. In addition, although in the line of previous studies mir-16 was strongly increased in IBS patients, the presence of *Blastocystis* can modulated circulating levels of mir-16. Taken together, it seems that *Blastocystis* may induce inflammation, disrupt the gut integrity, and modulate the gut environment to provide a stable condition for its colonization. However, due to the lack of metagenomics data to exclude the probable role of other variables, the role of *Blastocystis* in modulating the gut microbiota and the expression of miRNAs should be carefully interpreted.

## Data Availability

All generated data from the current study are included in the article.
